# Comparison between Dynamic Stabilization and Instrumented Fusion in the Treatment of Spinal Stenosis with Degenerative Lumbar Scoliosis

**DOI:** 10.1155/2022/9367106

**Published:** 2022-05-18

**Authors:** Lei Luo, Liehua Liu, Pei Li, Chen Zhao, Lichuan Liang, Fei Luo, Qiang Zhou, Yanhong Chen, Lang Fang

**Affiliations:** ^1^Department of Orthopedics, The Third Affiliated Hospital of Chongqing Medical University, Chongqing, China; ^2^Department of Orthopedics, Southwest Hospital, Army Medical University, Chongqing, China

## Abstract

**Objective:**

Posterior instrumented fusion is the most widely accepted surgical treatment for spinal stenosis with degenerative lumbar scoliosis (DLS). However, long fusion can affect daily activities due to lumbar stiffness. Dynamic stabilization has been introduced to overcome the drawbacks of fusion in recent years. This study aimed to compare the outcomes of dynamic stabilization (Dynesys system) with posterior instrumented fusion for the management of spinal stenosis with DLS.

**Methods:**

This study retrospectively reviewed 65 consecutive patients with spinal stenosis and DLS who were undergoing surgical treatment between January 2013 and December 2017. Among them, 34 patients (Dynesys group) had fenestration decompression and Dynesys stabilization, whereas 31 patients (fusion group) underwent posterior instrumented fusion. Clinical outcomes, radiographic data, and postoperative complications were compared between the two groups.

**Results:**

The mean number of fixed segments was 3.6 ± 0.9 in the Dynesys group and 4.2 ± 1.0 in the fusion group. Lower average values of operating time and blood loss were observed in the Dynesys group (*P* < 0.05). At an average follow-up of 42 months, there were no significant differences in the visual analog scale for the leg pain (VAS_leg_), the scoliosis Cobb's angle, and the lumbar lordosis between the two groups (*P* > 0.05). The visual analog scale for back pain (VAS_back_), oswestry disability index (ODI), and lumbar stiffness disability index (LSDI) scores of the Dynesys group were lower compared with the fusion group (*P* < 0.05). The range of motion (ROM) of implanted segments was significantly higher in the Dynesys group as compared to the fusion group (*P* < 0.05). The overall complications were less in the Dynesys group, but the difference was not statistically significant (*P* > 0.05).

**Conclusion:**

Both dynamic stabilization and instrumented fusion can improve the clinical outcomes of patients with spinal stenosis and mild DLS. Compared to instrumented fusion, dynamic stabilization has the advantages of less invasion and motion preservation.

## 1. Introduction

Degenerative lumbar scoliosis (DLS) is defined as a spinal deformity that develops during adulthood due to asymmetric degenerative changes of the disc, vertebral body, and facet joint, with a coronal Cobb measurement ≥10 [1]. It is a common disease in the middle-aged and elderly population [2]. In most patients with low back pain, the curve is likely to progress [3]. DLS is frequently associated with disc herniation, degenerative spondylolisthesis, and stenosis [4]. The symptoms are mainly low back pain, radicular pain, and neurogenic claudication. Patients with DLS often have some comorbidities such as hypertension, diabetes, or respiratory diseases. So the treatment should focus on alleviating symptoms and preventing further progression of scoliosis, rather than deformity correction [5].

When conservative treatment fails, surgical treatment should be considered. At present, surgical treatments mainly include simple spinal decompression and lumbar fusion with instrumentation [6]. Simple spinal decompression can relieve radicular pain in the lower limbs. However, decompression alone presents a poor long-term result which is related to the progression of the deformity. Compared with fusion surgery, simple spinal decompression is less effective in relieving low back pain and has a higher rate of postoperative symptom recurrence [7, 8]. Lumbar fusion with instrumentation is the most widely accepted surgical treatment. It can be divided into short fusion and long fusion. Long fusion was superior to short fusion in the correction of the Cobb angle, coronal imbalance, and lateral listhesis, but it is likely to increase perioperative complications [9]. In addition, long fusion can affect the activities of the lumbar spine, such as bending down, squatting, wiping after the stool, and so on [10].

**Figure 1:**
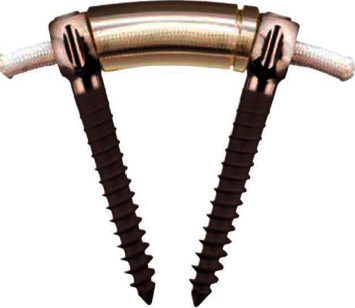
The dynesys system consists of titanium alloy screws, polyethylene terephthalate cords, and hollow cylinder polycarbonate urethane spacers.

In recent years, a dynamic stabilization system (Dynesys system, Figure 1) has been introduced to overcome the drawbacks of fusion. Several studies have shown that dynamic stabilization could stabilize the instrumented segments while preserving some mobility. Di Silvestre et al. reported that Dynesys dynamic stabilization in addition to laminectomy could lead to a significant symptom improvement and maintain enough stability to prevent progression of scoliosis and instability in elderly patients with degenerative lumbar scoliosis [11–13]. However, laminectomy damaged the posterior column structures of the lumbar spine which could increase the stress on the screws. Moreover, good lumbar lordosis was correlated with clinical symptoms [14], and significant lateral listhesis could lead to scoliosis progression [3]. But the Dynesys system has a limited ability to correct lumbar kyphosis [15] and lateral listhesis. Therefore, we treated DLS with fenestration decompression and Dynesys stabilization. Segments with obvious kyphosis and lateral listhesis underwent intervertebral fusion. This study aimed to compare the outcomes of dynamic stabilization with posterior instrumented fusion for the management of spinal stenosis with DLS.

## 2. Materials and Methods

### 2.1. Patients

This study retrospectively reviewed 65 consecutive patients with spinal stenosis and DLS who were undergoing surgical treatment between January 2013 and December 2017. Among them, 34 patients (Dynesys group) had fenestration decompression and Dynesys stabilization (five of the patients underwent single-segmental intervertebral fusion and other segments dynamic stabilization), and 31 patients (fusion group) underwent posterior instrumented fusion. This study has been approved by the Ethical Committee of the Third Affiliated Hospital of Chongqing Medical University (SKYW20190106). For this type of study, formal consent for the review of patients' images and medical records is not required. And the study was conducted under the ethical principles that have their origins in the Declaration of Helsinki and its subsequent amendments. Inclusion criteria were as follows: (1) age ≥40 years at the time of surgery; (2) coronal Cobb angle more than 10° but less than 30° before surgery, had an apex between L2 and L4; (3) combined with degenerative changes such as disc herniation, spinal stenosis, spondylolisthesis, etc.; (4) no improvement after 3-months of conservative treatment; (5) underwent the operation of dynamic stabilization (Dynesys, Zimmer Spine) or instrumented fusion surgery; and (6) with complete clinical and imaging data. Exclusion criteria were as follows: (1) a history of idiopathic scoliosis and scoliosis caused by tuberculosis, fracture, or other diseases; (2) a history of lumbar surgery; (3) sagittal imbalance of the spine; (4) severe osteoporosis (*T* value ≤ −2.5 with single or multiple fragility fractures or *T* value ≤ −3.0); and (5) with cervical spondylotic myelopathy, hip disease or other diseases that affected the judgment of therapeutic effect.

### 2.2. Surgical Procedure

All patients underwent general anesthesia in the prone position. Autologous blood transfusion was used during the operation.

**Figure 2:**
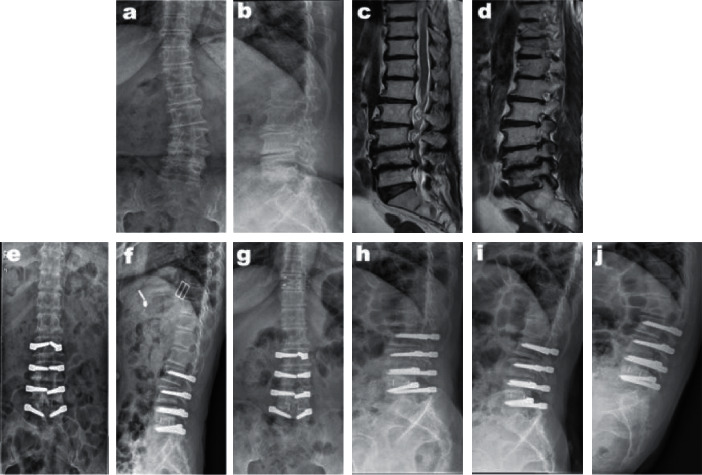
A 49-year-old woman had vertebral canal stenosis at L2–5 and left lateral recess stenosis at L4-5 with DLS (a–d). She underwent fenestration decompression at L3-4, TLIF at L4-5, and instrumentation at L2–5 using the Dynesys system. Postoperative radiographs showed scoliosis correction (e, f), the radiographs obtained 42 months after the operation showed motion preservation and no progression of scoliosis (g–j).

In the Dynesys group, interlaminar fenestration decompression was performed through the posterior median approach at the responsible levels. Both the spinal canal and lateral recess were decompressed, with the avoidance of excessive excision of the facet joint. When there were segments with recurvatum, lateral listhesis >12 mm, or foraminal stenosis which required removal of the facet joint, transforaminal lumbar interbody fusion (TLIF) was performed. Pedicle screws were inserted through the Wiltse approach under imaging control. The entry point was located at the junction of the lateral border of the superior articular process and the basilar part of the transverse process. The extent of fixation included decompressed segments and segments with instability, spondylolisthesis, and lateral listhesis, not to end at the apical vertebra. Then the patients' positions were modified to obtain the appropriate lumbar lordosis. The polycarbonate urethane spacer was cut according to the measured distance between the screws (distraction force of 1.0 N, longer on the concave side and shorter on the convex side). The spacer length was properly reduced in the fusion segment for intervertebral compression. The central cord and the spacer were then locked within the screw heads (Figure 2). Patients were treated with a lumbar belt for 3 weeks after surgery, while patients undergoing selective fusion were advised to wear a stiffer waist for 1 month.

**Figure 3:**
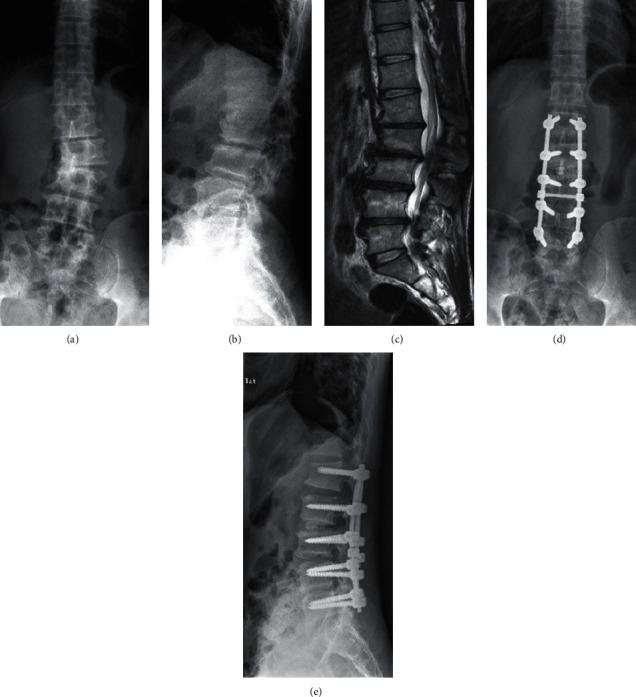
A 46-year-old man had spinal stenosis at L1–5 with DLS (a–c). She underwent fenestration decompression at L4-5, TLIF at L2-3, and posterolateral instrumented fusion at L1–5. The radiographs obtained 38 months after the operation showed stable scoliosis correction (d, e).

In the fusion group, the posterior median approach was used to expose the lamina and facet joints. Pedicle screws were positioned under imaging control. The horizontal vertebra was selected as the upper instrumented vertebra. L5 or S1 was selected as the lower instrumented vertebra. Curve correction was carried out by distraction on the concave side and compression on the convex side. When there were segments that needed discectomy, or lateral listhesis >12 mm, TLIF was performed. Posterolateral fusion was performed at other segments (Figure 3). The patients wore a brace for 3-months after surgery.

### 2.3. Clinical and Radiological Evaluation

Clinical outcomes were assessed through the visual analog scale (VAS) for back and leg pain, the oswestry disability index (ODI), and the lumbar stiffness disability index (LSDI) [16]. Operating time, blood loss, and complications were also documented. Posteroanterior, lateral, and dynamic radiographs with flexion and extension views were obtained preoperatively, postoperatively, and at the last follow-up. The radiological evaluation index included the lumbar scoliotic angle, the lumbar lordotic angle, and the range of motion (ROM). A “double halo sign” (radiolucent line around the implant >2 mm wide) on X-rays was defined as screw loosening.

### 2.4. Statistical Analysis

SPSS 16.0 software was used for statistical analysis. The chi-square test was used for categorical variables, whereas the *T*-test, two-factor repeated measures ANOVA, and covariance analysis were used for quantitative data. All significance tests were two-tailed. When *P* < 0.05, the difference was considered to be statistically significant.

## 3. Results

### 3.1. Perioperative Data and Complications

There were no statistically significant differences between the Dynesys group and the fusion group in age and sex (*P* > 0.05).

In the Dynesys group, the mean number of fixed segments was 3.6 ± 0.9. The mean operating time was 249.3 ± 60.7 minutes, while intraoperative blood loss was 713.2 ± 334.4 ml. One upper respiratory infection case and one pulmonary infection case were resolved after medical treatment. Poor wound healing occurred in 3 cases which were cured by secondary bedside suture under local anesthesia. One patient developed transitory radiating pain after surgery, which was relieved by medication. One patient developed muscle weakness of the lower extremity, which was recovered by neurotrophic therapy and functional exercise 1 month after surgery. The mean follow-up duration was 44 months (range, 36–78 months). Screw loosening was found on plain radiographs in one patient. There were no cases of incision infection, screw misplacement, screw breakage, or reoperation.

In the fusion group, the mean number of fixed segments was 4.2 ± 1.0. The mean operating time was 326.1 ± 55.0 minutes, while intraoperative blood loss was 1051.2 ± 427.7 ml. One atrial fibrillation case and one deep venous thrombosis case were resolved after medical treatment. Poor wound healing occurred in 2 cases which were cured by secondary bedside suture. One patient developed a surgical site infection, which was cured by antibiotics and debridement. Two patients developed transitory radiating pain after surgery, which was relieved by medication. One patient developed severe low back pain, which was alleviated by nonsteroidal anti-inflammatory drugs (NSAIDs). The mean follow-up duration was 39 months (range, 30–73 months). There were 3 cases of screw loosening, 1 case of proximal junctional kyphosis, and no cases of implant breakage or pseudarthrosis.

Lower average values of the number of fixed segments, operating time, and blood loss were observed in the Dynesys group than in the fusion group (*P* < 0.05). The incidence of complications was lower in the Dynesys group than in the fusion group (23.5% vs. 38.7%), but the difference was not statistically significant (*P*=0.185; Table 1).

### 3.2. Clinical Outcomes

The VAS and ODI scores improved significantly after the operation in both the groups (both *P* < 0.05). There was no statistically significant difference in VAS_back, leg_ and ODI scores between the two groups preoperative. However, the VAS_back_ scores at 6 months after surgery and the last follow-up, the ODI scores at the last follow-up were lower in the Dynesys group than that in the fusion group, and the difference was statistically significant (*P* < 0.05). There was no statistically significant difference in LSDI between the two groups before surgery (*P* > 0.05). The LSDI was significantly less in the Dynesys group as compared to the fusion group at the last follow-up (*P* < 0.05; Table 2).

### 3.3. Radiological Outcomes

#### 3.3.1. Scoliosis Cobb Angle

In the Dynesys group, the mean scoliosis Cobb angle was 15.6° ± 3.4° before surgery, 6.7° ± 2.8° after surgery, and 6.9° ± 2.4° at the last follow-up. In the fusion group, the mean scoliosis Cobb angle was 17.1° ± 3.0° before surgery, 6.3° ± 3.3° after surgery, and 6.2° ± 3.4° at the last follow-up. Compared with preoperative values, the scoliosis Cobb angle in both groups decreased significantly after surgery and at final follow-up (*P* < 0.05). In the Dynesys group, the difference between postoperative and last follow-up was not statistically significant (*P* > 0.05). There was no statistically significant difference between the two groups in preoperative, postoperative, and at the last follow-up (*P* > 0.05; Table 3).

#### 3.3.2. Lumbar Lordosis

In the Dynesys group, the mean lumbar lordosis was 32.2° ± 10.5° before the surgery, 34.8° ± 10.1° after the surgery, and 33.1° ± 9.3° at the last follow-up, with no significant differences (*P* > 0.05). In the fusion group, the mean lumbar lordosis was 29.4° ± 8.7° before the surgery, and increased to 36.9° ± 11.2° after the surgery, with statistically significant difference (*P* < 0.05). There was no statistically significant difference between the two groups in lumbar lordosis before the surgery, after the surgery, and at the last follow-up (*P* > 0.05; Table 3).

#### 3.3.3. Range of Motion

There were no significant differences in the mean ROM values of the L1-S1 levels and the implanted segments between the two groups preoperatively. However, ROM at L1-S1 and implanted segments of the Dynesys group were significantly higher than that of the fusion group (28.8° ± 7.3° vs. 8.3° ± 1.6° and 11.7° ± 4.3° vs. 0.80° ± 0.26°, respectively) at the final follow-up (*P* < 0.05; Table 3).

## 4. Discussion

Although scoliosis correction is not the main goal of surgery, it is essential to prevent scoliosis from further aggravation of conditions. Simotas et al. followed up 49 patients with lumbar spinal stenosis receiving conservative treatment for 3 years and found that lumbar scoliosis was one of the reasons for poor results [17]. Instrumented fusion has advantages in scoliosis correction and maintaining lumbar lordosis. However, there are some disadvantages, such as long operating time, excessive blood loss, and a high incidence of perioperative complications [18]. In addition, most DLS patients are elderly, usually suffering from chronic diseases simultaneously. Therefore, a surgery less invasive than instrumented fusion would be considered in treating DLS. Fenestration decompression, Dynesys stabilization, and selective intervertebral fusion were used in this study. The results showed that both the operating time and blood loss were significantly less in the Dynesys group compared to the fusion group. This is likely because Dynesys stabilization reduced the number of instrumented segments and the operation procedures of the facet joints resection, clearance of intervertebral space, and preparation of the bone graft bed. The operating time and blood loss are risk factors for perioperative complications [19]. Our data showed the incidence of complications in the Dynesys group was lower than that in the fusion group (23.5% vs. 38.7%), although the difference was not statistically significant.

In this study, VAS_back, leg_ and ODI scores improved in both the groups at the last follow-up compared with preoperative scores, indicating that both methods were effective for the treatment of DLS. However, VAS for low back pain and ODI scores showed better improvement in the Dynesys group than in the fusion group. It may be due to the Wiltse approach with less disturbance to lumbar dorsal muscles and nonfusion surgery allowing for early functional rehabilitation.

In recent years, patients have become increasingly concerned with the function of the lumbar spine after long segments fusion [20]. Some patients complained about the inconvenience caused by lumbar stiffness in daily life, such as wearing shoes, taking a shower, and wiping after stool [21]. Robert et al. designed the lumbar stiffness disability index (LSDI) scale to evaluate the limitations of daily activities caused by lumbar stiffness [16]. The LSDI consists of 10 questions with higher scores representing greater difficulty in performing activities of daily living because of lumbar stiffness. In this study, the LSDI scale increased significantly in the fusion group after surgery, while there was no statistical difference between preoperative and the last follow-up scores in the Dynesys group. Furthermore, ROM at L1-S1 and implanted segments of the Dynesys group were significantly higher than that of the fusion group at the final follow-up. The results suggest that dynamic stabilization with the Dynesys system does not impair the function of the lumbar spine in treating DLS.

In the Dynesys group, scoliosis was well corrected from 15.6° preoperatively to 6.7° postoperatively, and 6.9° at the last follow-up, without significant loss of correction. Although scoliosis correction was better in the fusion group than in the Dynesys group, the difference was not statistically significant. This suggests that dynamic stabilization can correct mild scoliosis and prevent scoliosis progression.

Lateral listhesis is an important radiographic parameter that affects the clinical symptoms of patients with DLS. Moderate to severe lateral listhesis (equal to or more than 6 mm) demonstrated more severe back pain than mild lateral listhesis [22]. Intervertebral recurvatum is also an important factor affecting long-term clinical efficacy. Intervertebral recurvatum or insufficient lordosis causes increased angular motion at the adjacent levels [23]. The loss of lumbar lordosis was also closely related to the clinical symptoms of low back pain [14]. Therefore, significant lateral listhesis and intervertebral recurvatum should be corrected. However, elastic spacers and connectors in the Dynesys system have an inadequate ability to correct lateral listhesis and intervertebral recurvatum. A study has shown that hybrid stabilization could better preserve the lordosis of instrumented segments [24]. In this study, transforaminal lumbar interbody fusion was performed at the segments with obvious lateral listhesis and intervertebral recurvatum. In addition, the patients' position was modified to obtain the appropriate lumbar lordosis before stabilization. The results showed that lumbar lordosis was well maintained or improved after the operation, and the effect was comparable to that of fusion surgery. Dynesys is unable to correct the obvious sagittal imbalance of the spine, so this study did not involve these cases.

Screw loosening in dynamic stabilization has been a concern. Patients with degenerative scoliosis are mostly elderly, often combined with osteoporosis, making this concern more prominent. There were big differences in the incidence of screw loosening with the Dynesys system reported in the literature (range 0–73.5%) [25]. Yu et al. compared radiographic outcomes of Dynesys and posterior lumbar interbody fusion (PLIF) for the treatment of multisegment degenerative disc disease with a minimum follow-up of 3 years and confirmed no significant difference in the incidence of screw loosening between the two groups [26]. Wu et al. analyzed 658 screws in 126 patients with an average age of 60.4 years, 31 screws (4.7%) in 25 patients (19.8%) were shown to have loosened during an average follow-up period of 37.0 months. All 25 patients with screw loosening were asymptomatic, and in 6 (24%) osseous integration was demonstrated on later follow-up [27]. In this study, the rate of screw loosening was lower in the Dynesys group compared with that in the fusion group and previous reports for several reasons. Firstly, the fenestration decompression has less damage to the stability of the lumbar spine. Secondly, the screw placement was improved. The pedicle screws were inserted as deep as possible under imaging control. Thirdly, elastic spacers and connectors can disperse stress on the implants. Finally, patients with severe osteoporosis were excluded from this study.

## 5. Conclusions

This study demonstrates that both Dynesys dynamic stabilization and instrumented fusion can improve clinical outcomes of patients with spinal stenosis and DLS. Compared to instrumented fusion, dynamic stabilization has advantages of less invasion and motion preservation. Dynesys stabilization can also correct mild lumbar scoliosis and prevent progression of the curve. Nonetheless, larger and longer-term studies are needed to establish the long-term safety and efficacy of dynamic stabilization.

## Figures and Tables

**Table 1:** Comparison of demographic and clinical characteristics of 2 groups of patients who underwent dynamic stabilization or fusion.

characteristics	Dynesys group (*n* = 34)	Fusion group (*n* = 31)	Statistic	*P* value
Age (years)	62.8 ± 11.6	58.2 ± 12.2	*t* = 1.558	0.124
Sex (male/female)	11/23	9/22	*χ*2 = 0.084	0.772
Segments (*n*)	3.6 ± 0.9	4.2 ± 1.0	*t* = 2.546	0.013
Operating time (minutes)	249.3 ± 60.7	326.1 ± 55.0	*t* = 5.327	0.001
Blood loss (ml)	713.2 ± 334.4	1051.2 ± 427.7	*t* = 3.566	0.001
Complication *n*, (%)	8, (23.5)	12, (38.7)	*χ*2 = 1.754	0.185

**Table 2:** Clinical outcomes

	Dynesys group (*n* = 34)	Fusion group (*n* = 31)	*P* value
^a^VAS_back_			
Pre op	5.3 ± 1.7	5.5 ± 1.9	0.502
6 months postoperative	2.2 ± 1.2	3.2 ± 1.1	0.009
Last follow-up	2.1 ± 1.0	2.8 ± 1.1	0.008
^b^VAS_leg_			
Pre op	5.6 ± 1.5	5.8 ± 1.2	0.367
6 months postoperative	1.6 ± 0.4	1.7 ± 0.4	0.952
Last follow-up	1.8 ± 0.5	1.9 ± 0.4	0.583
ODI (%)			
Pre op	64.9 ± 16.8	63.2 ± 18.3	0.550
6 months postoperative	31.2 ± 10.3	32.9 ± 11.2	0.670
Last follow-up	21.8 ± 9.2	30.5 ± 10.1	0.001
LSDI (%)			
Pre op	21.6 ± 9.4	23.2 ± 10.3	
Last follow-up	24.9 ± 9.7	40.4 ± 10.4	0.001

^a^VAS_back_ VAS scores for back pain, ^b^VAS_leg_ VAS scores for leg pain.

**Table 3:** Radiological outcomes.

	Dynesys group (*n* = 34)	Fusion group (*n* = 31)	*P* value
Scoliosis (°)			
Pre op	15.6 ± 3.4	17.1 ± 3.0	0.055
Postoperative	6.7 ± 2.8	6.3 ± 3.3	0.802
Last follow-up	6.9 ± 2.4	6.2 ± 3.4	0.386
Lumbar lordosis (°, L1-S1)			
Pre op	32.2 ± 10.5	29.4 ± 8.7	0.427
Postoperative	34.8 ± 10.1	36.9 ± 11.2	0.175
Last follow-up	33.1 ± 9.3	35.8 ± 9.9	0.073
ROM (°, L1-S1)			
Pre op	36.3 ± 11.9	32.1 ± 12.7
Last follow-up	28.8 ± 7.3	8.3 ± 1.6	0.001
ROM (°, implanted segments)			
Pre op	21.9 ± 8.6	23.3 ± 10.2
Last follow-up	11.7 ± 4.3	0.80 ± 0.26	0.001

## Data Availability

The data used to support the findings of this study are included within the article.
